# Enhancing TextGCN for depression detection on social media with emotion representation

**DOI:** 10.3389/fpsyg.2025.1612769

**Published:** 2025-08-26

**Authors:** Huimin Mao, Qing Han

**Affiliations:** School of Medical Technology and Information Engineering, Zhejiang Chinese Medical University, Hangzhou, China

**Keywords:** graph convolutional networks, depression detection, emotion representation, social media, pre-trained language models, mental health, psychology

## Abstract

**Background:**

Depression, also known as depressive disorder, is a pervasive mental health condition that affects individuals across diverse backgrounds and demographics. The detection of depression has emerged as a critical area of research in response to the growing global burden of mental health disorders.

**Objective:**

This study aims to augment the performance of TextGCN for depression detection by leveraging social media posts that have been enriched with emotional representation.

**Methods:**

We propose an enhanced TextGCN model that incorporate emotion representation learned from fine-tuned pre-trained language models, including MentalBERT, MentalRoBERTa, and RoBERTaDepressionDetection. Our approach involves integrating these models into TextGCN to capitalize on their emotional representation capabilities. Furthermore, unlike previous studies that discard emoticons and emojis as noise, we retain them as individual tokens during preprocessing to preserve potential affective cues.

**Results:**

The results demonstrate a significant improvement in performance achieved by the enhanced TextGCN models, when integrated with embeddings learned from MentalBERT, MentalRoBERTa, and RoBERTaDepressionDetection, compared to baseline models on five benchmark datasets.

**Conclusion:**

Our research highlights the potential of pre-trained models to enhance emotional representation in TextGCN, leading to improved detection accuracy, and can serve as a foundation for future research and applications in the mental health domain. In the forthcoming stages, we intend to refine our model by incorporating more balanced and targeted data sets, with the goal of exploring its potential applications in mental health.

## 1 Introduction

Depressive disorder, commonly referred to as depression, is a widespread mental health condition that affects individuals of all backgrounds and demographics. According to global statistics, approximately 280 million people worldwide were affected by depression in 2019, with a significant proportion of this population comprising children and adolescents, numbering 23 million (World Health Organization, 2025). Characterized by a persistent decline in mood or a marked diminution of pleasure in activities over an extended period, depressive disorder has far-reaching consequences that permeate various aspects of life, including academic performance, workplace productivity, and interpersonal relationships with family, peers, and the broader community. Furthermore, depression poses a substantial risk of suicidal behavior, which remains a leading cause of mortality among individuals aged 15–29, underscoring the imperative need for comprehensive support and intervention strategies. There is an imperative need to address this condition, as a substantial number of individuals suffering from mental disorders, particularly depression, remain underserved by adequate care. Despite the availability of proven prevention and treatment strategies, a considerable proportion of individuals with mental health issues continue to lack access to comprehensive care (Altamura et al., 2008; Bukh et al., 2013; Fekadu et al., 2022; Carey et al., 2014; Yang et al., 2022). Untreated depression can have a detrimental impact on the disease progression and prognosis, ultimately resulting in suboptimal outcomes. Consequently, early detection of depression is crucial, as it not only optimizes treatment efficacy but also alleviates the burden on healthcare systems, reduces reliance on specialized providers, and mitigates the associated social stigma (Uddin et al., 2022).

The detection of depression has emerged as a critical area of research in response to the growing global burden of mental health disorders. Traditionally, the diagnosis of depression has relied heavily on the International Classification of Diseases (ICD) and the Diagnostic and Statistical Manual of Mental Disorders (DSM), which are susceptible to subjective bias and the clinician’s diagnostic expertise (Rubin, 2018). However, recent initiatives have sought to revolutionize the field by harnessing advancements in artificial intelligence (AI) and machine learning to enhance early and accurate detection. The integration of AI in mental health care is increasingly acknowledged by practicing psychiatrists, who foresee its pivotal role in shaping the future of care (Doraiswamy et al., 2020; Han and Zhao, 2025). These cutting-edge tools leverage a diverse array of data sources, including clinical interviews, speech patterns, electroencephalograms, wearable device metrics, and electronic health records, to identify subtle indicators of depression (Squires et al., 2023). Researchers anticipate discovering a disparity in social media activity between individuals with typical social behaviors and those with depression. The extensive usage of social media platforms has led to a significant increase in users sharing their thoughts and personal experiences, which can provide valuable insights into the detection of depression (Islam et al., 2018). Longitudinal data from social media platforms has been recognized as a highly valuable resource, offering broad self-disclosure opportunities (Balani and De Choudhury, 2024). By leveraging this data, numerous studies have proposed depression detection models based on traditional machine learning approaches. However, manual feature extraction from user posts is often necessary, utilizing various technologies such as Linguistic Inquiry and Word Count (LIWC), N-grams, Term Frequency-Inverse Document Frequency (TF-IDF), and Bag of Words (BOW) (Guntuku et al., 2017). The integration of these technologies enables researchers to develop detection models using a range of traditional machine learning algorithms, including Naive Bayes, Logistic Regression, Support Vector Machines (SVM), and Random Forest, among others (Philip Thekkekara et al., 2024; Manna and Nakai, 2019).

Despite notable progress achieved by traditional machine learning algorithms in the field of depression detection, several limitations persist. The manual construction of features necessitates substantial expertise and domain knowledge, as well as considerable effort to identify relevant features for training (Malhotra and Jindal, 2022). In contrast, deep learning techniques can automatically extract features from raw text vectors and provide abstract summaries of information, whereas traditional approaches often rely on shallow semantic features or statistical text models. Recent studies have leveraged deep learning algorithms, including Convolutional Neural Networks (CNN), Recurrent Neural Networks (RNN), and algorithms incorporating attention components and transformer-based architectures such as BERT (Devlin et al., 2018), to improve depression detection outcomes based on social media text data (Orabi et al., 2018). CNN models have demonstrated exceptional proficiency in extracting local features and patterns from text data through the application of convolutional operations. Conversely, Graph Neural Networks (GNNs) have shown remarkable efficacy in capturing global semantic relationships and structured dependencies within text data, which can be effectively represented as graphs. The Text Graph Convolutional Network (TextGCN) (Liang et al., 2018) leverages the strengths of both CNN and GNN approaches, thereby enabling the integration of word-level and document-level semantic information in a comprehensive manner. This innovative methodology facilitates the effective comprehension of complex human emotions, particularly those associated with depression, rendering TextGCN a valuable tool for this application. However, the current implementation of TextGCN relies on one-hot vector representations of individual words or documents, without the incorporation of pre-trained word embeddings or external knowledge (Liang et al., 2018). This limitation underscores the need for the development of more advanced representation techniques that can accurately capture the intricate emotional nuances embedded within text data.

Building a model to identify depression through social media data typically involves a multi-step approach: data acquisition, data preprocessing, the creation of word representations, model training, and model validation. Emotion representation is a critical component of natural language processing (NLP), as it enables models to accurately capture the emotional nuances of human language, thereby enhancing their comprehension of emotional dimensions. The application of various techniques, such as word embeddings (e.g., Word2Vec, GloVe) and contextualized embeddings (e.g., BERT, RoBERTa), has been widely adopted to encode emotional cues in text, resulting in improved performance in tasks like text classification (Aydoğan and Karci, 2020), sentiment analysis (Rezaeinia et al., 2019), emotion detection, and depression detection (Lara et al., 2021; Lestandy and Abdurrahim, 2023). Recent research has integrated emotion representation into depression detection models, aiming to enhance accuracy and contribute to advancements in mental health research and practice. For example, models like TextGCN with Emotion Graph Representation have demonstrated promise in abstracting both explicit and implicit emotional signals associated with mental health, showcasing the potential of emotion representation in depression detection (Cabral et al., 2024).

This study concentrates on augmenting the performance of TextGCN, subsequently developing an improved model based on TextGCN for depression detection by harnessing social media posts enriched with emotion representation. To achieve this goal, we using the pre-trained emotion representation to replace the basic input for TextGCN. And we compared the different contextual representations generated by various pre-trained large language models for the depression detection models. Combining the embedding technique and the TextGCN approach, we propose our models as the depression detection tool, incorporating posts from social media platforms such as Twitter, Reddit, and web forums.

Our approach and key contributions can be summarized as follows.

1. We explored the applicability of the emotion representations in depression detection, contributing to the future work in this field.
2. We propose a new framework for depression detection with a higher accuracy and availability, which achieved the better performance on three publicly available social media posts depression detection datasets. The construction contains two parts, the pre-trained emotion representation and the Textual Graph Convolutional Networks classification (TextGCN).
3. Comprehensive experiments across three datasets yield the following key findings: Utilizing pre-trained word embeddings significantly outperforms using one-hot vectors as input to TextGCN. Models leveraging RoBERTa-based embeddings consistently outperform those based on BERT embeddings. Fine-tuning further enhances the performance of both BERT-and RoBERTa-based models compared to their base versions. Models trained on longer texts achieve better results than those trained on shorter texts.

The remainder of this paper is organized as follows: Section 2 presents the methods has employed for depression detection, particularly the approaches like Textual Graph Convolutional Networks classification (TextGCN) and emotion representation. Section 3 introduces the specific technical methods in our model. The whole experimental content is described in Section 4, from datasets to model etc. Results are discussed in Section 5. Lastly, Section 6 conclude the paper.

## 2 Related works

### 2.1 Depression detection

Depression is a pervasive mental health disorder that affects millions of individuals worldwide, often without being consciously acknowledged. It can lead to a significant diminishment of interest in everyday activities, potentially culminating in suicidal ideation. Traditionally, depression diagnosis is based on standardized clinical criteria, which encompasses current symptomatology and medical history (Smith et al., 2013). The integration of big data and machine learning algorithms presents an effective and efficient means of automating depression detection, providing valuable support to healthcare professionals and patients alike. At its core, classic depression detection constitutes a classification problem, wherein the distinction between healthy and depressed individuals or the prediction of severity is paramount (Dinkel et al., 2019).

Researchers have conducted an in-depth examination of behavioral indicators, encompassing facial expressions, speech patterns, and other multi-modal signals. For example, Shin et al. (2022) leveraged clinical interview transcripts to train a machine learning model for the detection of depression and suicidal risk. This study employed the Naive Bayes classifier, yielding an area under the curve (AUC) of 0.905, sensitivity of 0.699, and specificity of 0.964 for diagnosing depression. A study conducted by Hadžić et al. (2024) explored the potential of artificial intelligence, specifically machine learning, as a diagnostic tool for depression in clinical interviews. This investigation focused on the performance of a BERT-based natural language processing (NLP) model, which achieved an accuracy of 0.71. In contrast, an untrained GPT-3.5 model demonstrated superior performance, achieving an accuracy of 0.88.

The integration of social media into mental health studies presents a novel avenue for accessing data from a broader population beyond traditional clinical settings. This expansion enables researchers to gather insights from a wider subset of individuals, including those who may not have been previously accounted for in clinical studies (Cabral et al., 2024). Younger generations have been found to be more inclined to express suicidal ideation on social media, rather than disclosing it to healthcare professionals or family members (John et al., 2018). In contrast to clinical data, social media data offers several advantages, including ease of access, richer content, and lower concealment potential (Malhotra and Jindal, 2022). Several studies have demonstrated the potential of social media data in detecting mental health issues. For instance, Govindasamy and Palanichamy (2021) utilized Twitter data to develop a depression detection model, employing a combination of Naïve Bayes and NBTree classifiers to achieve optimal results. Ayyalasomayajula’s (2024) investigation into the impact of linguistic features on depression detection through Reddit posts yielded impressive outcomes, with an exceptional *F*_1_-score achieved using a TF-IDF-based logistic regression model on the same dataset. This research highlights the potential of social media data in mental health studies, and its ability to outperform traditional methodologies. The findings of this study contribute to the growing body of evidence on the use of social media data in mental health research, and underscore the importance of exploring this avenue in future studies.

The application of deep learning technologies, excluding traditional machine learning approaches, has been increasingly employed in the detection of depression. Orabi et al. (2018) leveraged Convolutional Neural Networks (CNNs) and Recurrent Neural Networks (RNNs) to identify individuals exhibiting signs of mental illness, utilizing limited amounts of unstructured data sourced from Twitter. Their CNNWithMax models demonstrated a higher accuracy of 87.957%, with optimized embedding, and were found to outperform RNN models in depression detection. In contrast, Mihov et al. (2022) utilized heterogeneous graph convolution to develop a depression detection model, MentalNet, by representing users’ social circles, including analysis of user interactions and the intimacy of user contacts. The framework achieved excellent performance on Twitter data. Liu (2024) integrated XLM-RoBERTa and TextGCN to propose a depression clinical detection model based on data from Twitter, Reddit, and Weibo. XLM-RoBERTa was employed to extract semantic insights from multilingual text, while TextGCN was leveraged to acquire knowledge of the multilingual text structure. The study demonstrated that the incorporation of TextGCN significantly enhanced the performance of the model. TextGCN represents a pioneering approach, wherein the entire corpus is represented as a heterogeneous graph, enabling the simultaneous construction of word and document representations via Graph Neural Networks (GNNs). This novel framework outperforms state-of-the-art text classification methods, without relying on pre-trained word embeddings or external knowledge, and has been successfully evaluated on a range of benchmark datasets. In contrast, prior studies (Lin et al., 2021; Han et al., 2022; Li et al., 2023) have sought to augment GCN models by incorporating pre-trained representations or integrating external knowledge.

### 2.2 Emotion representation

Embeddings are numerical vector representations of text data, enabling efficient processing and analysis by computers. This technique is commonly employed in natural language processing tasks, such as sentiment analysis, and serves as a crucial component in deep learning methods. Embeddings have been developed in various forms, including character, word, and sentence levels, and have been widely explored in existing studies (Zhang and Han, 2025).

Research has demonstrated the potential of word embeddings in detecting depression on social media platforms, such as Reddit. A study by Lestandy and Abdurrahim (2023) investigated the impact of word embedding dimensions on the detection of depression using a Bidirectional Long Short-Term Memory (BiLSTM) model in conjunction with Word2Vec and GloVe methods. The findings highlight the efficacy of combining word embeddings with Recurrent Neural Network (RNN) technology for depression identification, with the Word2Vec approach yielding significant advantages. Couto et al. (2022) proposed a framework for early depression detection by analyzing changes in language use on social media, utilizing temporal word embeddings. The proposed method employed two temporal word embedding models: TWEC, based on word2vec, and DCWE, built on pre-trained language models like BERT. The experimental results demonstrated that the DCWE model outperformed most participants in the CLEF eRisk tasks of 2017 and 2018, achieving top performance in ERDE5 and ERDE50 metrics, and even surpassing state-of-the-art methods in some cases. In a study by Goswami et al. (2024) explored depression detection by combining embeddings extracted by BERT or RoBERTa with a classifier RNN, whose models achieved a validation accuracy of 99.9%. Hong et al. (2022) proposed a novel approach to node attributes within a Graph Neural Network (GNN)-based model, where node-specific embeddings are captured for each word in the vocabulary to measure the severity of depression symptoms. Xin et al. (2024) compared the suitability of various pre-trained language models, including BERT, MentalBERT, MentalLongformer, Llama 2-7B, and Llama 3-8B, across three different segmentations of interview transcripts. The findings revealed that LLM embeddings facilitated efficient classification of outcomes in qualitative studies on adolescent depression, and confirmed their potential in future detection and treatment of depression. Bucur’s (2024) study compared two transformer-based embedding methods, MentalRoBERTa and an MPNet variant, for representing Reddit social media posts and BDI-II questionnaire responses. The results showed that the model designed for semantic search performed better than the mental health pre-trained model in embedding tasks, highlighting the performance differences of various embedding methods in depression symptom detection.

Briefly, numerous studies explore the model integrating emotion representation and machine learning or deep learning technology for depression detection, achieving certain results. And GNN can improve the models’ ability of representation generation. In the study, we attempt to detect depression from different social media platforms and websites specifically designed to support patients with depression to further expand the scope of our model using; we apply the deep learning method that can use those measures for the detection of individuals who are suffering from depression; we propose a novel computational framework that can improve the accuracy for automatic depression detection.

## 3 Methods

### 3.1 Overview

**Figure 1:**
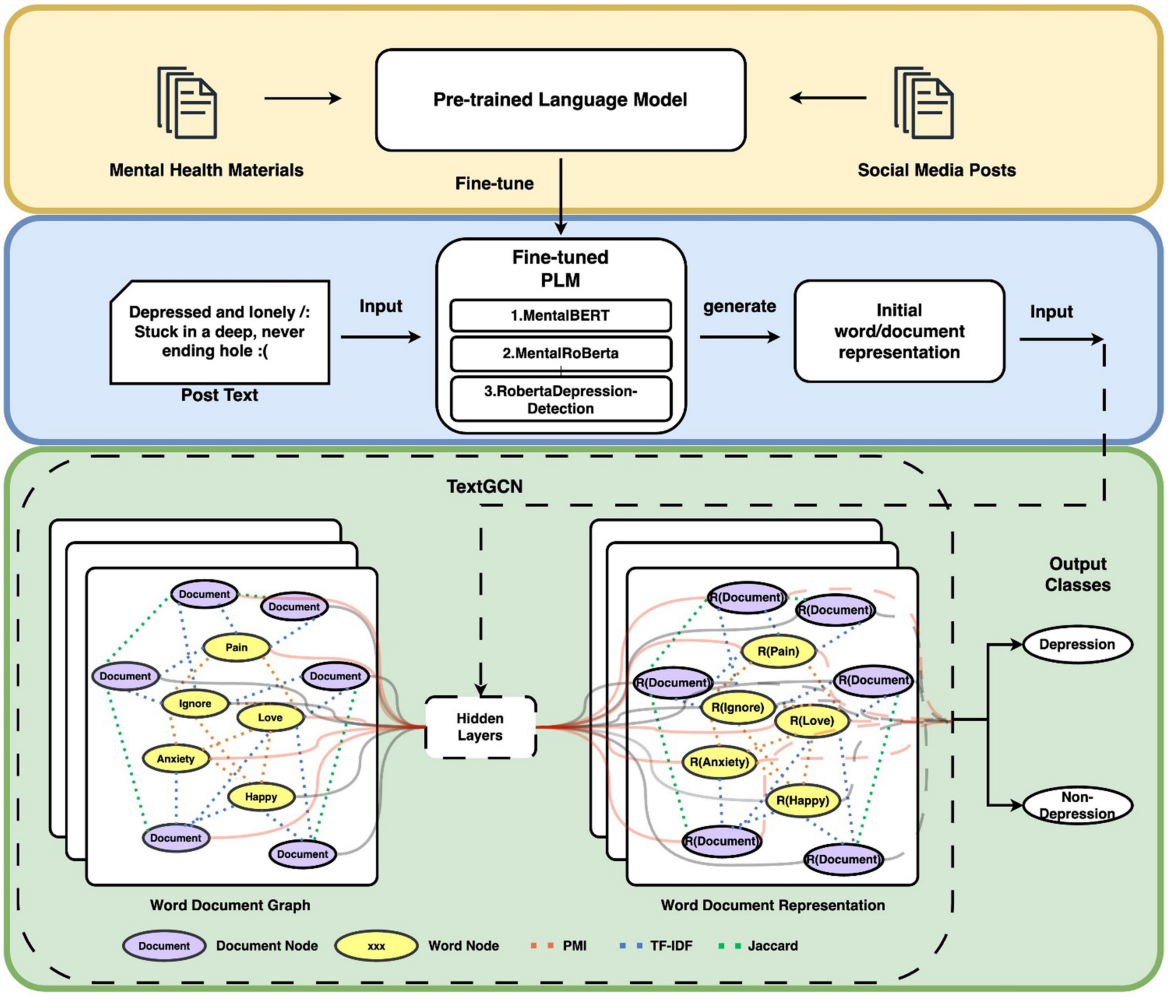
Overview of our model’s architecture.

We enhance the TextGCN (Liang et al., 2018) by incorporating emotion representation to investigate the trend of depression detection in social media. At its core, TextGCN posits that a text corpus can be effectively represented as a graph, G=(V,E,A) , where V comprises a set of word and document nodes. The edges E consist of word-word connections $E_{w_{i}w_{j}}$ , word-document relationships $E_{w_{i}d_{j}}$ , and document-document connections $E_{d_{i}d_{j}}$ . $A\in R^{n\times n}$ is the graph adjacency matrix, representing the weights between nodes in the graph. Figure 1 illustrates the architecture of our depression detection classification model enhanced by emotion representation.

To inform our model, we leverage pre-trained models that have been domain-adapted or fine-tuned using mental health materials or other relevant information to generate emotion embeddings.

We construct a text graph, where nodes represent words and documents in the corpus, and edges represent their interconnections. Our fine-tuned pre-trained language model generates emotion representations as initial weights for the TextGCN model. The constructed graph is then passed through two layers of Graph Convolutional Networks (GCN) for depression detection tasks.

### 3.2 TextGCN

#### 3.2.1 Node-edge construction

##### 3.2.1.1 Node construction

The node set V comprises all documents and unique words within the corpus. Assuming there are D documents and M unique words in the corpus, the total number of nodes is ∣V∣=n=D+M . Building upon the work of Han et al. (2022), we leverage pre-trained embeddings to enhance the TextGCN model. For example, we could utilize BERT to generate the initial node representation, where the learned vector for the [CLS] token serves as the basis for initializing each document node. Each document $D_{d}$ is processed through the BERT, yielding a sequence representation $B_{D_{d}}$ for that document. For example, a document $D_{d}$ such as “Sunny feels happy” results in $B_{D_{d}}$ as $B_{D_{d}}^{\left[\mathrm{CLS}\right]}B_{D_{d}}^{\text{Sunnny}}B_{D_{d}}^{\text{feels}}B_{D_{d}}^{\text{happy}}B_{D_{d}}^{\left[\mathrm{SEP}\right]}$ after representation learning.

We utilize the [CLS] representation $B_{D_{d}}^{\left[\mathrm{CLS}\right]}$ as the node embedding for $D_{d}$ . Next, we construct the document context by aggregating all documents containing the word $W_{m}$ , represented as $D_{W_{m}}$ . Subsequently, a min-pooling operation is applied to all BERT representations $B_{D_{d}}^{W_{m}}$ of the word $W_{m}$ across the document collection.

##### 3.2.1.2 Edge construction

Inspired by the work of Han et al. (2022), we utilize the all co-occurring relations between every two types of nodes.

The set of edges is $E=\{E_{w_{i}w_{j}},E_{w_{i}d_{j}},E_{d_{i}d_{j}}\}$ , where $E_{w_{i}w_{j}}$ represents Pointwise Mutual Information, (PMI), where $E_{w_{i}d_{j}}$ represents Term Frequency-Inverse Document Frequency (TF-IDF), and $E_{d_{i}d_{j}}$ represents Jaccard similarity. Finally, we get the graph adjacency matrix A as follows.

$$A_{\mathrm{ij}}=\{\begin{array}{c} {\mathrm{PMI}}_{\mathrm{ij}},i,j\;\mathrm{are}\;\text{words};\mathrm{PMI}>0\\ \mathrm{TF}-{\mathrm{IDF}}_{\mathrm{ij}},i\;\text{is word},j\;\text{is document}\\ {\text{Jaccard}}_{\mathrm{ij}},i,j\;\mathrm{are}\;\text{documents}\\ 0,\text{otherwise} \end{array}$$

#### 3.2.2 GCN for text classification

The central concept of GCN (Shin et al., 2022) is to consolidate information from a node’s neighboring nodes via graph convolution operations. Each layer within the GCN disseminates information according to the following formula (Equation 1):


(1) $$H^{\left(l+1\right)}=f(H^{\left(l\right)},A)=\sigma (\hat{A}H^{\left(l\right)}W^{\left(l\right)})$$

where $H^{\left(l\right)}$ is the node feature matrix at the l -th layer, and $H^{\left(0\right)}$ is the initial node feature matrix. $\hat{A}={\tilde{D}}^{-\frac{1}{2}}\tilde{A}{\tilde{D}}^{-\frac{1}{2}}$ is the normalized symmetric adjacency matrix, where $\tilde{A}=A+I$ , and I is the identity matrix used to add self-connections. $\tilde{D}$ is the degree matrix, where ${\tilde{D}}_{\mathrm{ii}}=\sum_{j}{\tilde{A}}_{\mathrm{ij}}$ . $W^{\left(l\right)}$ is the trainable weight matrix at the l -th layer. σ is a non-linear activation function, typically ReLU.

We evaluate our models through a depression detection task. This approach offers a more straightforward and feasible implementation compared to alternative methods. We utilize a diverse set of initial word-document representations, including one-hot encoding, three large language models (two fundamental and three pre-trained), and three models specifically designed for mental health classification and depression detection. To construct co-occurrence information, we leverage pre-defined methods. Following the construction of the text graph, we apply a two-layer Graph Convolutional Network (GCN) architecture. The output of the first layer is processed through a Rectified Linear Unit (ReLU) activation function, while the output of the second layer is routed through a softmax function to facilitate classification. The specific formulas are as follows (Equations 2, 3):


(2) $$H^{\left(1\right)}=\text{ReLU}(\hat{A}H^{\left(0\right)}W^{\left(0\right)})$$

(3) $$Z=\text{softmax}(\hat{A}H^{\left(1\right)}W^{\left(1\right)})$$

The final classification loss function is the cross-entropy loss (Equation 4):


(4) $$L=-\sum_{d\in y_{D}}\sum_{f=1}^{F}Y_{\mathrm{df}}\text{In}Z_{\mathrm{df}}$$

where $y_{D}$ is the set of labeled document nodes, whose documents were collected from social media. F is the output feature dimension which is equal to 2 in depression detection, Y is the label indicator matrix, $Y_{\mathrm{df}}=1$ indicates that document d belongs to category f . $Z_{\mathrm{df}}$ is the probability output by GCN that document d belongs to category f . Our framework is designed to ultimately yield superior results in tasks related to depression detection.

### 3.3 Emotion representation

MentalBERT and MentalRoBERTa are pre-trained language models tailored variants of the BERT and RoBERTa architectures, respectively, specifically designed for processing mental health-related text data. Ji et al. (2021) employed a domain-adaptive pretraining approach, wherein they continued to fine-tune the models on a mental health corpus after initializing them with general pre-trained weights to adapt the models to the distinctive characteristics of mental health-related text. The mental health corpus utilized in this study comprises 13,671,785 sentences extracted from mental health-related posts on Reddit, sourced from various subreddits, such as “r/depression,” “r/SuicideWatch,” and “r/Anxiety.” The pre-training process was conducted using Huggingface’s Transformers library, with the Masked Language Modeling (MLM) task employed, consistent with BERT and RoBERTa, where MentalBERT utilized static masking, whereas MentalRoBERTa employed dynamic masking. The experimental results demonstrate that MentalBERT and MentalRoBERTa outperform baseline models across a range of mental health detection tasks, with notable superiority in depression detection.

Additionally, RoBERTaDepressionDetection (Hugging Face, 2025) is a fine-tuned variant of the twitter-roberta-base model, specifically designed for depression detection tasks. This model is initialized with the twitter-roberta-base model, a RoBERTa-base variant that has undergone extensive pre-training on approximately 58 million tweets. The model was further refined through fine-tuning by the developer, who utilized a corpus from the LT-EDI 2022 shared task, a dataset aimed at detecting depression through social media text analysis. In accordance with the task’s requirements, the model was trained to categorize text into three distinct classes: moderate depression, severe depression, and non-depression. The training dataset comprises 53,909 sentences, with 7,884 sentences classified as non-depression, 36,114 sentences categorized as moderate depression, and 9,911 sentences labeled as severe depression. The average document length for each class is 4, 6, and 11 sentences, respectively, with corresponding average sentence lengths of 78, 100, and 140 words. During the fine-tuning process, the developer employed a Multilayer Perceptron (MLP) as the classifier and utilized the Adam optimizer.

Compared to baseline models, MentalBERT, MentalRoBERTa, and RoBERTaDepressionDetection significant advantages in generating emotion representations. These models achieve superior performance in capturing domain-specific semantic features through domain-adaptive pretraining or task-specific fine-tuning, thereby enhancing their accuracy. MentalBERT and MentalRoBERTa demonstrate particular strength in producing embeddings that effectively represent domain-specific terminology, emotional expressions, and contextual subtleties. In contrast, RoBERTaDepressionDetection excels in decoding depression-related linguistic patterns, such as mood fluctuations and symptom descriptions, present in social media texts. The domain-specific training employed in these models enables them to adapt more robustly to nuanced mental health expressions, including self-negation and anxiety, as well as common noise patterns found in social media texts, such as abbreviations and informal syntax. Furthermore, the emotion representations generated by these models can be directly applied to downstream tasks with reduced fine-tuning requirements, and have been shown to outperform general models in mental health detection tasks.

### 3.4 Depression detection classification

We conduct an evaluation of the enhanced TextGCN model, which leverages emotion representation learned from fine-tuned pre-trained language models to perform depression detection in a post-based framework. This approach allows for the benefits of post-based classification to be realized without the necessity of incorporating social media components, such as historical posts. By constructing a graph of words and documents, we then pass it through a two-layer Graph Convolutional Network (GCN) designed for depression detection. Utilizing categorical cross-entropy as the loss function, we employ single-label classification to achieve accurate depression detection outcomes.

## 4 Results

### 4.1 Datasets

**Figure 2:**
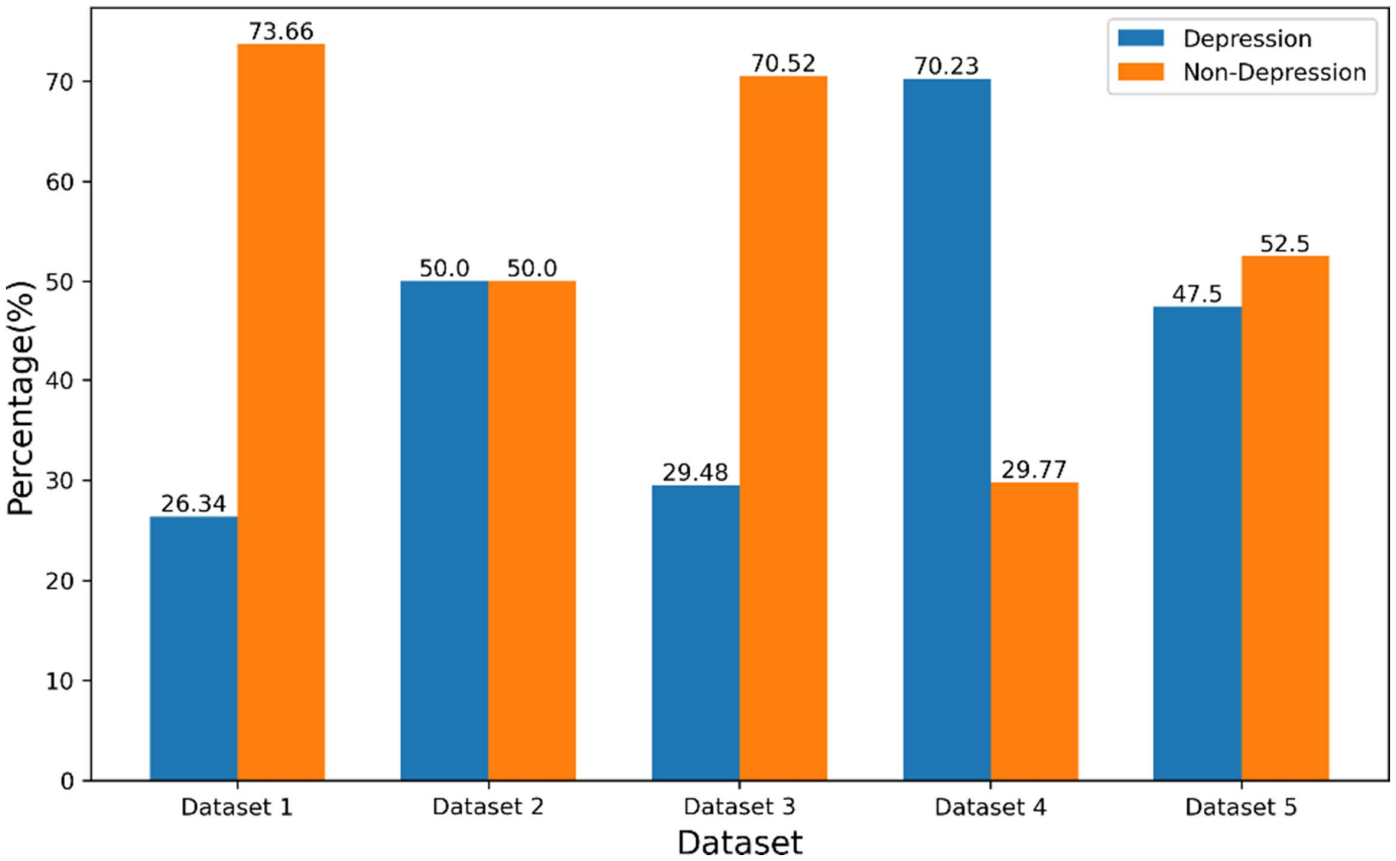
Class distribution for each dataset.

We employed a diverse set of publicly available datasets to assess the efficacy of our model. The statistics and distribution of categories for each dataset are summarized in Table 1, while Figure 2 provides a visual representation of the categorical distribution.

**Table 1:** Dataset statistics.

Dataset No.	Original dataset name	Source	Number of posts	Positive/Negative	Average text length
Dataset 1	The Twitter depression dataset	Twitter	3,200	843/2,357	96
Dataset 2	The multiple languages twitter datasets (English-language)	Twitter	1,000	500/500	133
Dataset 3	The identifying-depression datasets	Web Forums	1,323	390/933	1,101
Dataset 4	The identifying-depression datasets	Reddit	1841	1293/548	1,123
Dataset 5	The identifying-depression datasets	Reddit & Web Forums	2,821	1340/1,481	1,133

The Twitter depression dataset (MacAvaney et al., 2021), which served as the basis for the practice dataset for the CLPsych 2021 competition, was constructed by collecting tweets related to depression, removing hashtags, and annotating them using binary classes (depression, D, 26.34%, and non-depression, ND, 73.66%). We denoted this dataset as “Dataset 1” and the majority of the dataset consists of brief, emotive posts with emojis.

In addition to the Twitter depression dataset, we also utilized the multiple languages Twitter datasets proposed by Cha et al. (2022). These datasets comprise 921 k tweets from Korean users, 10 M tweets from English users, and 15 M tweets from Japanese users. Each language-specific dataset was labeled using a depression lexicon collected from previous studies on detecting depression on social media, with depression and non-depression categories assigned a binary classification (1 for depression, 0 for non-depression). In this study, we focused on the English-language dataset, which primarily consists of brief posts with emojis. This was denoted as “Dataset 2”.

Furthermore, we drew upon the Identifying-Depression Datasets collected by Pirina and Çöltekin (2024), which aggregated social media data from Reddit and web forums to identify depression. We annotated each post according to the labeling provided by the authors. The datasets were divided into three sub-datasets, denoted as “Dataset 3,” “Dataset 4,” and” “Dataset 5”. The “Dataset 3” and “Dataset 4” consisted of long posts with emojis, sourced from web forums and Reddit, respectively. The “Dataset 5” combined content from both sources, providing a comprehensive dataset for depression detection.

### 4.2 Evaluation metrics

To assess the overal performance of our depression detection models, we employ four widely used evaluation metrics: Accuracy, Precision, Recall, and class *F*_1_-score. These metrics provide a comprehensive evaluation of the models’ ability to correctly identify depressive cases.

Accuracy is a fundamental metric that quantified as the proportion of correctly predicted instances over the total number of instances. It is defined as follows (Equation 5):


(5) $$\text{Accuracy}=\frac{\mathrm{TP}+\mathrm{TN}}{\mathrm{TP}+\mathrm{TN}+\mathrm{FP}+\mathrm{FN}}$$

where TP (true positive) represents the count of depressed users who are accurately identified. TN (true negative) signifies the count of non-depressed users who are accurately identified. FP (false positive) indicates the count of non-depressed users who are inaccurately identified. FN (false negative) denotes the count of depressed users who are inaccurately identified.

Precision is calculated as the ratio of true positives among all positive predictions. It is defined as follows (Equation 6):


(6) $$\text{Precision}=\frac{\mathrm{TP}}{\mathrm{TP}+\mathrm{FP}}$$

A high precision indicates that the model produces fewer false positives, which is crucial in clinical applications where wrong detection could lead to unnecessary interventions.

Recall is defined as the proportion of true positives that are correctly identified. It measures the ability of the model to correctly identify all actual depressive cases. It is defined as follows (Equation 7):


(7) $$\text{Recall}=\frac{\mathrm{TP}}{\mathrm{TP}+\mathrm{FN}}$$

A high recall value ensures that most depressive cases are correctly identified, ensuring that individuals in need receive appropriate attention.

The *F*_1_-score, representing the harmonic mean of Precision and Recall, serves as a balanced measure of both, providing a holistic assessment of a model’s performance. It is defined as follows (Equation 8):


(8) $$F_{1}-\text{Score}=\frac{2\times \text{Precision}\times \text{Recall}}{\text{Precision}+\text{Recall}}$$

A higher *F*_1_-score reflects a better trade-off between Precision and Recall, making it a valuable metric for evaluating depression detection models.

### 4.3 Experiment setup

**Figure 3:**
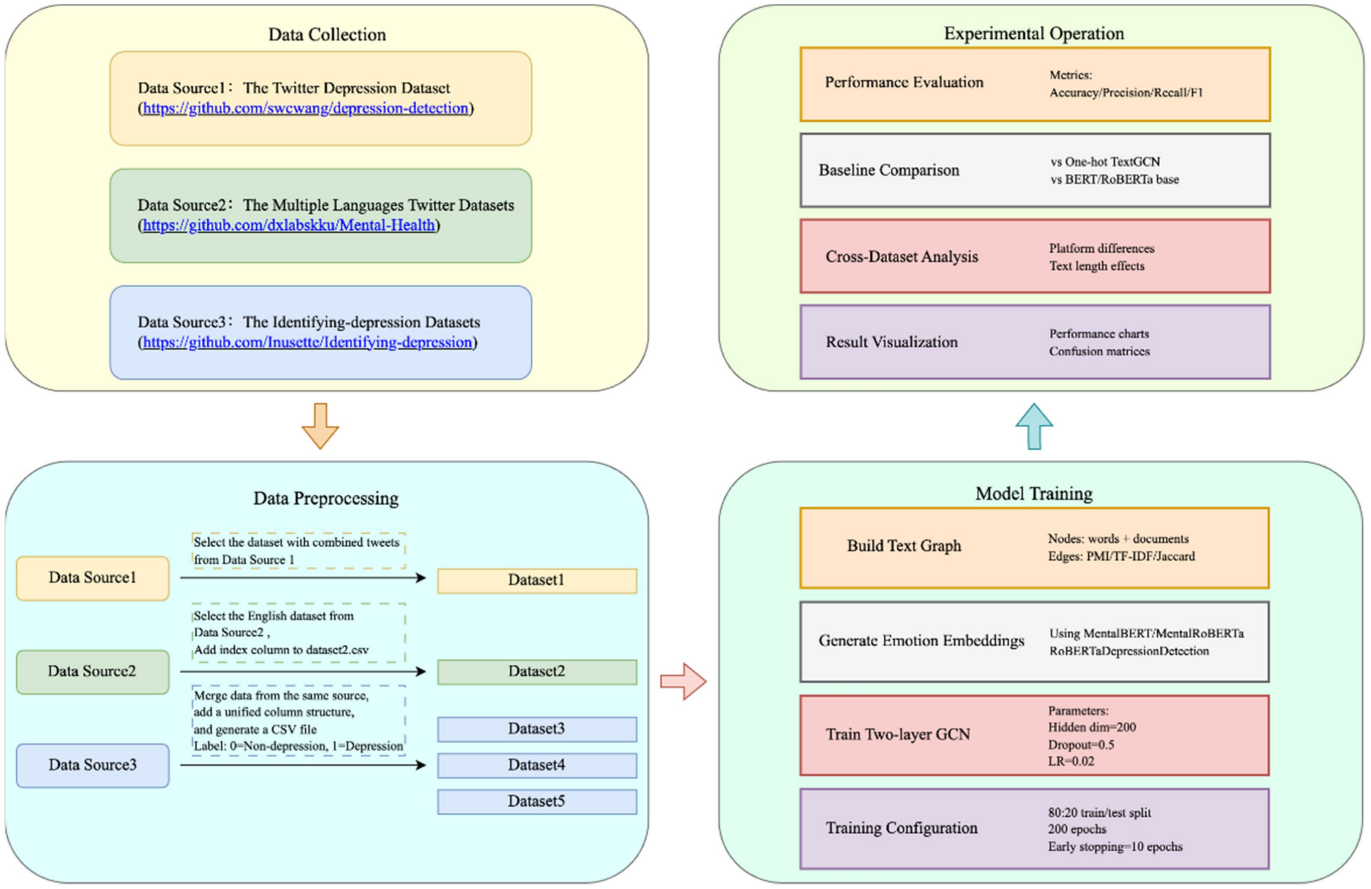
The diagram of this experiment.

We propose an advanced TextGCN framework that leverages diverse emotion representations, derived from pre-trained language models, as input. To substantiate its efficacy, we conduct a comprehensive evaluation of its performance across five publicly available datasets in the depression domain, in comparison to the basic TextGCN with one-hot encoding. Figure 3 shows the diagram of this experiment.

In our experiments, we assess the performance of our proposed system in relation to five pre-trained language models (PLMs) that utilize transformation-based approaches. The BERT model (Devlin et al., 2018) is a transformer-based pre-trained language representation model that has achieved state-of-the-art results on numerous NLP tasks through its next sentence prediction and masked language modeling capabilities. RoBERTa (Liu et al., 2019), a variant of BERT, builds upon the same training setup but incorporates dynamic masking, expands the training dataset, and increases the batch size to enhance its performance. In contrast, MentalBERT, MentalRoBERTa, and RoBETaDepressionDetection are domain-specific pre-trained language models, tailored to the depression detection task, and are built upon either BERT or RoBERTa, respectively. These models have demonstrated improved performance in mental health detection tasks and depression detection tasks through fine-tuning on mental health-related data.

We employed a 80:20 train/test split to train our model, with a training duration of 200 epochs and an early stopping criterion of 10 epochs, utilizing the Adam optimizer for training. To further ensure the robustness and generalizability of the proposed method, we also conducted K-fold cross-validation, where the dataset was partitioned into K subsets and each subset was used as the test set exactly once. We specified the following hyperparameters: a hidden dimension size of 200, a dropout rate of 0.5, a learning rate of 0.02, and a configuration of two GCN layers. Hyperparameter tuning was conducted using Optuna, a hyperparameter optimization framework. The optimized hyperparameters included the number of hidden layers (L = {2, 3, 4, 5}), hidden layer dimensions (H = {100, 200, 300, 400, 500}), dropout rates (dr = {0.01, 0.05, 0.1, 0.5}), learning rates (lr = {0.01, 0.02, 0.03, 0.04, 0.05}), and weight decay values (wd = {0, 0.005, 0.05}). We acknowledge the potential for information leakage resulting from the inclusion of test nodes in the graph during training. This issue may inadvertently introduce indirect supervision signals and affect the validity of performance evaluation. In future work, we plan to explore alternative graph construction strategies or transductive-to-inductive adaptations to mitigate such leakage and ensure a more rigorous experimental setup.

### 4.4 Experiment results

Our model evaluation protocol involves a depression detection task, which serves as a benchmark for assessing the performance of our proposed approach. Table 2 provides a comparative analysis of our model’s results with the initial TextGCN model, which relies solely on a one-hot vector input without incorporating pre-trained emotion representations or external knowledge. For each evaluation metric (Accuracy, Precision, Recall, *F*_1_-Score), *p*-values were calculated by paired t-tests comparing each optimized model to the baseline (One Hot). Significant improvements are highlighted where *p* < 0.05.

**Table 2:** The results of the enhanced TextGCN with emotion representation learned from pre-trained language models.

Dataset No.	Models	Accuracy	Precision	Recall	*F*_1_-score
Dataset 1	One Hot	0.7878	0.6947	0.3704	0.4805
	BERT	0.7929	0.6485	0.4913^*^	0.5590^*^
	RoBERTa	0.7932	0.6254	0.5653^**^	0.5932^**^
	MentalBERT	0.7834	0.6115	0.5195^*^	0.5616^*^
	MentalRoBERTa	0.7939	0.6252	0.5714^**^	0.5965^**^
	RoBERTaDepressionDetection	0.7940	0.6255	0.5712^**^	0.5966^**^
Dataset 2	One Hot	0.7727	0.7755	0.7747	0.7747
	BERT	0.7835	0.7973	0.7684	0.7811
	RoBERTa	0.7876^*^	0.7929	0.7884^*^	0.7891^*^
	MentalBERT	0.7872^*^	0.7987	0.7763	0.7857
	MentalRoBERTa	0.7896^*^	0.7910	0.7960^*^	0.7922^*^
	RoBERTaDepressionDetection	0.7869^*^	0.7791	0.8074^*^	0.7927^*^
Dataset 3	One Hot	0.8429	0.6725	0.8845	0.7638
	BERT	0.8119	0.8205^*^	0.9229^*^	0.8685^*^
	RoBERTa	0.8596^*^	0.8836^**^	0.9118^*^	0.8974^**^
	MentalBERT	0.8279	0.8369^*^	0.9258^*^	0.8788^*^
	MentalRoBERTa	0.9020^**^	0.9162^**^	0.9407^**^	0.9281^**^
	RoBERTaDepressionDetection	0.8958^**^	0.9037^**^	0.9465^**^	0.9245^**^
Dataset 4	One Hot	0.7957	0.9223	0.7851	0.8479
	BERT	0.8328^*^	0.9449	0.8177^*^	0.8762^*^
	RoBERTa	0.8402^*^	0.9528	0.8210^*^	0.8813^*^
	MentalBERT	0.8434^*^	0.8832	0.9041^**^	0.8935^**^
	MentalRoBERTa	0.8676^**^	0.9496	0.8638^*^	0.9043^**^
	RoBERTaDepressionDetection	0.8654^**^	0.9437	0.8646^*^	0.9024^**^
Dataset 5	One Hot	0.8986	0.9462	0.9172	0.9314
	BERT	0.7854	0.8743	0.8329	0.8530
	RoBERTa	0.8532	0.9233	0.8766	0.8992
	MentalBERT	0.8252	0.9238	0.8355	0.8772
	MentalRoBERTa	0.9104^*^	0.9593^*^	0.9193	0.9388^*^
	RoBERTaDepressionDetection	0.9096^*^	0.9578^*^	0.9197	0.9382^*^

Statistical tests were conducted using paired *t*-tests between each model and the baseline. Statistically significant improvements were denoted by ^*^p < 0.05, ^**^p < 0.01, and ^***^p < 0.001.

Firstly, pre-trained models have demonstrated superior performance compared to One-Hot encoding across various tasks, particularly in terms of recall and *F*_1_-score metrics. In dataset 1, the RobertaDepressionDetection model achieved a statistically significant improvement of 24.162% in *F*_1_-score and 0.787% in accuracy over One-Hot encoding, showcasing its enhanced semantic capture capabilities. Similarly, in dataset 3, the MentalRoBERTa model outperformed One-Hot encoding by 21.511% in *F*_1_-score and 7.012% in accuracy, further substantiating its superiority in domain adaptation. In dataset 5, pre-trained models experienced some decline in performance, but MentalRoBERTa retained marginal gains, indicating its robustness in handling diverse datasets.

Secondly, Roberta has been found to outperform BERT in most tasks, with notable improvements in *F*_1_-score and accuracy. In dataset 3, RoBERTa surpassed BERT by 3.328% in *F*_1_-score and 5.875% in accuracy, highlighting its enhanced performance in specific tasks. Furthermore, in dataset 5, RoBERTa achieved a 8.6325% higher accuracy and a 5.416% higher *F*_1_-score compared to BERT, validating its superiority across various datasets.

Thirdly, the domain-adaptive pre-trained models (MentalBERT and MentalRoBERTa) and the depression detection fine-tuned model (RobertaDepressionDetection) have demonstrated enhanced performance compared to their base counterparts (BERT/RoBERTa) in specific tasks.

MentalBERT has consistently outperformed the basic BERT model in mental health-related tasks, as evident in dataset 5, where it achieved a notable 5.068% accuracy boost and a 2.837% *F*_1_-score enhancement. However, in dataset 1, MentalBERT’s accuracy was lower than BERT, indicating a trade-off between domain specialization and generalization. Conversely, MentalRoBERTa has consistently outperformed RoBERTa, as seen in dataset 3, where it achieved a 4.933% accuracy increase and a 3.421% *F*_1_-score improvement. Additionally, RoBERTaDepressionDetection outperformed RoBERTa in dataset 3, with a 3.020% higher *F*_1_-score, but only a 0.456% increase in accuracy in dataset 2, highlighting the dependence on data quality.

Additionally, as shown in Table 2, the optimized models consistently outperformed the baseline across all metrics. Statistically significant improvements were denoted by ^*^p < 0.05, ^**^p < 0.01, and ^***^p < 0.001.

## 5 Discussion

The results of our experiments provide comprehensive insights into the performance of various fine-tuned pre-trained language models across different datasets, highlighting the importance of dataset quality and emotion representation. Our research results underscore the need for a multifaceted approach to optimization. From a dataset perspective, it is essential to consider platform-specific linguistic conventions, emoji semantics, class balance, and other relevant factors in the design phase. In model development, we recommend incorporating cutting-edge architectural innovations, domain adaptation techniques, and hybrid training strategies to optimize emotion representation for enhancing model performance. This analysis synthesizes empirical evidence to inform both technical implementation and data strategy decisions in the context of depression detection research.

### 5.1 Impact of text length and contextual richness

**Figure 4:**
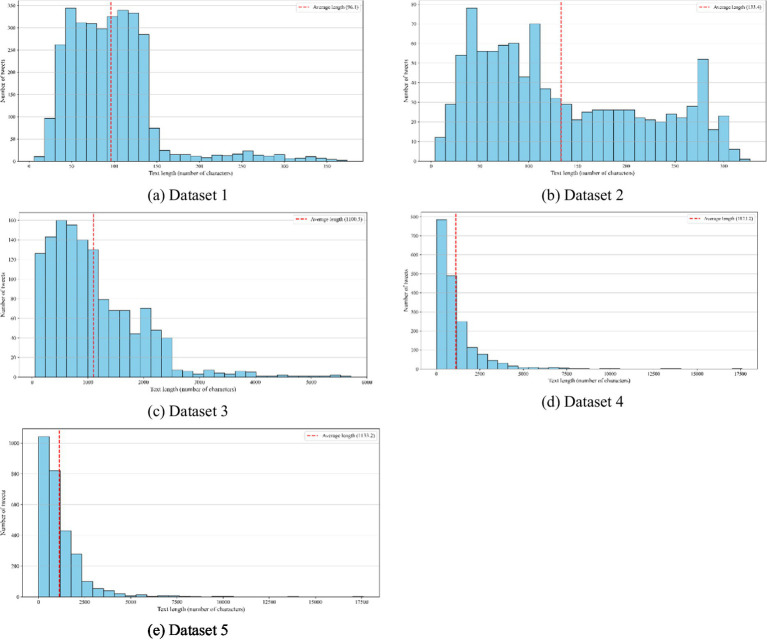
Distributions of text length across the five datasets. Subplots correspond to: **(a)** Dataset 1; **(b)** Dataset 2; **(c)** Dataset 3; **(d)** Dataset 4; and **(e)** Dataset 5.

Table 3 provides the text length statistics for the five datasets, whereas Figure 4 visualizes the distribution of text lengths within these datasets. The average text length varies considerably across the datasets. Dataset 1 has a mean length of 96 words with a standard deviation of 53, ranging from 6 to 374 words. Dataset 2 shows a slightly longer average text length of 133 words (SD = 85), with lengths ranging from 4 to 327 words. In contrast, Datasets 3 to 5 contain substantially longer texts. Dataset 3 has a mean length of 1,101 words (SD = 817), with a minimum of 52 words and a maximum of 5,717. Dataset 4 exhibits a higher variability, with a mean of 1,123 words and a standard deviation of 1,328, spanning from 11 to 17,601 words. Dataset 5 is similar to Dataset 4, with a mean length of 1,133 words (SD = 1,148), and the same minimum and maximum text lengths ranging from 11 to 17,601.

**Table 3:** Text length statistics for the five datasets.

Dataset No.	Text length statistics			
	Mean	Std.	Min.	Max.
Dataset 1	96	53	6	374
Dataset 2	133	85	4	327
Dataset 3	1,101	817	52	5,717
Dataset 4	1,123	1,328	11	17,601
Dataset 5	1,133	1,148	11	17,601

These statistics indicate that the datasets differ significantly in text length, from short texts in Datasets 1 and 2 to much longer and more variable texts in Datasets 3 to 5. Consistent with the findings of Wongkoblap et al. (2021) models trained on longer posts (e.g., Dataset 3, Dataset 4, and Dataset 5) exhibited superior performance, likely attributable to richer contextual signals. Social media texts often convey nuanced expressions spanning multiple sentences, requiring the preservation of complete contextual integrity. Future research should explore hybrid methods to balance context preservation with computing efficiency.

### 5.2 Impact of emoticons

In previous studies, emoticons were often regarded as noise and removed during mental health detection or emotion analysis (Maghraby and Ali, 2022; Samee et al., 2023). In this study, however, we preserve emoticons and emojis by treating them as individual tokens for contextualization. Meanwhile, emoticon recognition technology is emerging. For example, mapping emojis to affect-aware embeddings has shown significant potential for leveraging their affective value (Chen et al., 2018). Although this study did not further utilize emojis beyond tokenization, future research could incorporate recognition techniques to better exploit the affective information carried by emoticons in social media data, without introducing additional noise.

### 5.3 Impact of imbalanced data

**Figure 5:**
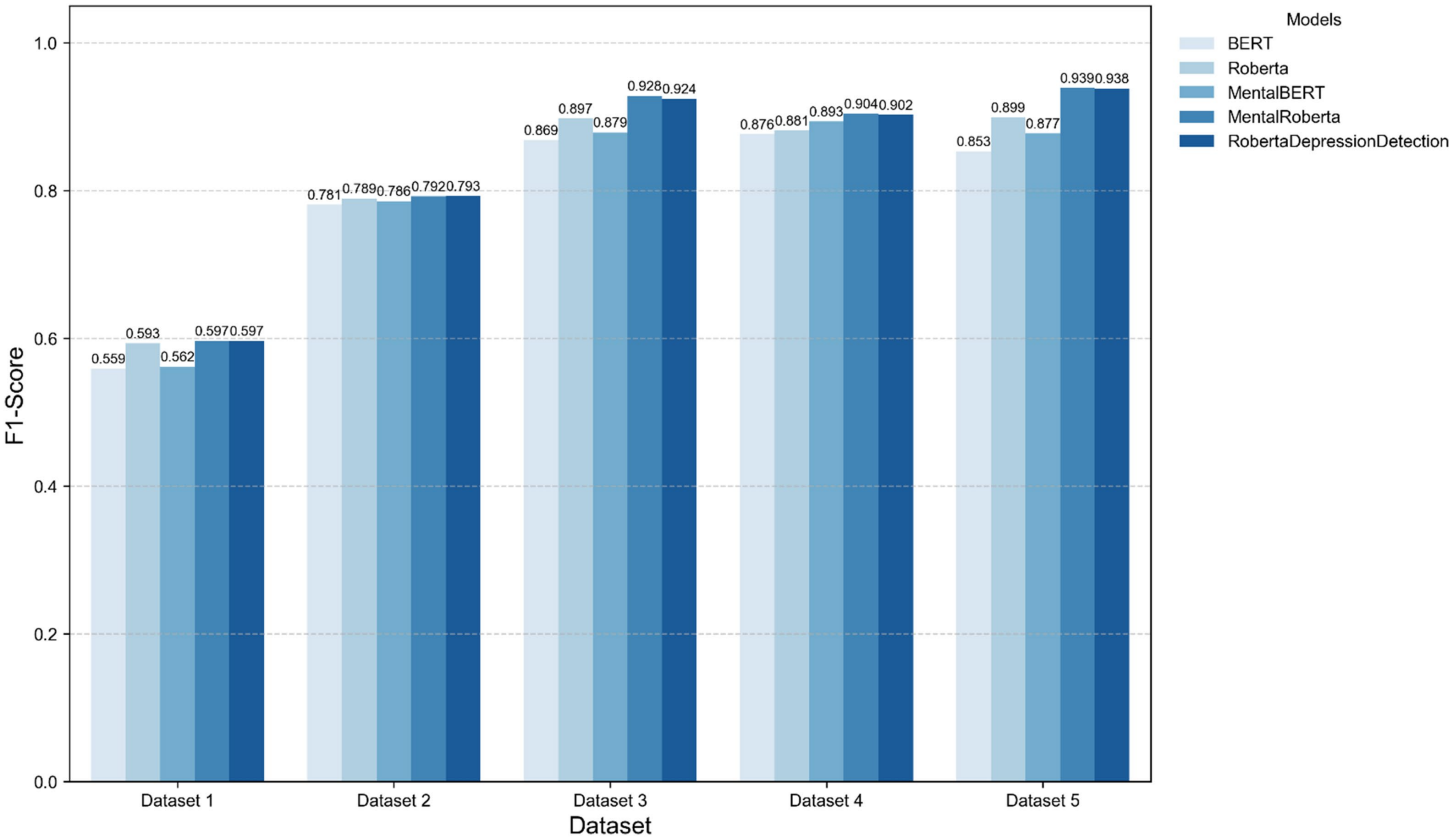
Performance comparison on TextGCN models enhanced with five different emotion representation.

Balanced datasets (e.g., *F*_1_-score in Dataset2 & Dataset5) outperformed skewed distributions, corroborating class imbalance as a critical challenge in mental health detection (Adel et al., 2024). Notably, domain-specific models like MentalRoBERTa partially mitigated imbalance effects (Dataset 4 *F*_1_-score + 2.61%), suggesting that domain-aware feature learning compensates for distributional skewness. Figure 5 illustrates the performance comparison on TextGCN models enhanced with emotion representation learned from five different pre-trained language models.

### 5.4 Architectural advantages of RoBERTa

RoBERTa and RoBERTa-based models outperform BERT and BERT-based models in most tasks. This suggests that RoBERTa’s unique features, such as dynamic masking and large-batch training, make it more adaptable to multitasking requirements. This aligns with clinical NLP studies where RoBERTa variants excel at detecting subtle symptom patterns (Lewis et al., 2020).

### 5.5 Domain adaptation vs. task-specific fine-tuning

MentalRoBERTa’s success highlights the effect of domain adaptation, whereas RobertaDepressionDetection’s data-dependent performance reveals the limitations of task-specific fine-tuning in low-resource settings. A promising direction involves hybrid strategies like staged training, which has achieved state-of-the-art results in biomedical NLP (Beltagy et al., 2019).

### 5.6 Domain specificity and data homology

MentalBERT and MentalRoBERTa exhibit superior performance on datasets comprising Reddit posts, given their pre-training data originates from Reddit, while RobertaDepressionDetection excels on Twitter datasets. The results reflect cross-platform generalization challenges arising from platform-specific linguistic norms, user language and demographic characteristics (Liu et al., 2019). Adversarial domain adaptation, as explored in prior work, may mitigate such discrepancies (Sun et al., 2019). But all pre-trained models outperform than the foundational ones. This emphasizes the pioneering exploratory value of the proposed framework in the field of early depression detection.

## 6 Conclusion

Due to the importance of protection of individual privacy, detecting depression through social media posts is a challenging task. Recent advancements in computer technology have enabled the development of novel algorithms for early depression detection, with some research focusing on enhancing contextual representations to improve model performance. Our work introduces a novel approach to text-based depression detection by incorporating pre-trained emotion representations into a graph-based classification model, TextGCN. This model effectively captures the semantic and syntactic relationships between words and documents, and we evaluate its performance on a depression detection task. Our results demonstrate that TextGCN with emotion representations outperforms baseline models across five datasets, with pre-trained models in specific domains showing superior performance compared to their baseline counterparts. This study proposes a novel framework for early depression detection, which has the potential to improve individual well-being and reduce the societal and economic burdens associated with untreated mental disorders.

Although emoticons and emojis were preserved as individual tokens to maintain contextual integrity, they were not incorporated into the embedding-level representations. Future work could explore mapping these elements to affect-aware embeddings to better leverage their emotional signals without introducing noise. Further more, our current approach is limited by the inability to dynamically generate input for TextGCN, relying solely on the pre-trained model’s embeddings. This limitation may hinder achieving enhanced prediction performance, which could be realized through concurrent training with both the pre-trained language models and TextGCN. To address this limitation, we intend to develop better pre-trained language models with more balanced and targeted data in the future, with the goal of achieving improved prediction performance.

## Data Availability

The original contributions presented in the study are included in the article/Supplementary material, further inquiries can be directed to the corresponding author.
